# Supplementary material for: The critical role of magma degassing in sulphide melt mobility and metal enrichment
Source: Nat Commun. 2022 Apr 29;13:2359. doi: 10.1038/s41467-022-30107-y (PMC9054852; doi:10.1038/s41467-022-30107-y)
Supplement: Supplementary file 1 — Supplementary Information [file 41467_2022_30107_MOESM1_ESM.pdf]

The critical role of magma degassing in sulphide melt mobility and metal enrichment

Iacono-Marziano G.[1], Le Vaillant M.[2], Godel B.M. [2], Barnes S.J. [2], and Arbaret L.[1]
[1]Institut des Sciences de la Terre d'Orléans, UMR 7327 CNRS-Université d'Orléans-BRGM, 45071 Orléans, France, Giada.Iacono@cnrs-orleans.fr
[2]CSIRO, Mineral Resources, Kensington, WA 6151, Australia

Supplementary Tables 1, 2, 3, 4, Supplementary Figures 1 and 2, and Supplementary text

Supplementary Table 1: Glass compositions normalised to 100

Supplementary Table 2: Sulphide droplet compositions

Supplementary Table 3: Olivine composition

Supplementary Table 4. HRXCT statistics

Supplementary Figure 1: Average Cu and Ni content of the sulphide melt versus the amount of fluid phase

Supplementary Text : Calculations of variations in Pt and Pd contents with silicate/sulphide mass ratio R

Supplementary Figure 2: Pt and Pd contents of Noril'sk-Talnakh ores

Supplementary Table 1. Glass compositions normalised to 100

Sample	n	SiO_2 [a] (wt%)	TiO_2 [a] (wt%)	Al_2O_3 [a] (wt%)	FeO^{tot} [a] (wt%)	MgO [a] (wt%)	CaO [a] (wt%)	Na_2O [a] (wt%)	K_2O [a] (wt%)	S [a] (wt%)	Total [b] (wt%)	Ni [c] (ppm)	Cu [c] (ppm)	Co [c] (ppm)	V [c] (ppm)
Starting glass															
SG*	39	48.0 (4)	0.88 (9)	15.6 (2)	10.8 (5)	11.4 (1)	10.8 (1)	1.61 (7)	0.60 (6)	bdl	99.7 (8)	708 (4)	931 (22)	73 (1)	201 (2)
Without decompression															
MLV01	41	50.0 (7)	0.9 (1)	17.1 (6)	7.2 (4)	10.1 (2)	11.5 (4)	1.80 (8)	0.62 (6)	0.5 (2)	95.2 (9)	19 (3)	11 (1)	19 (1)	210 (5)
MLV02	28	49.4 (3)	0.91 (9)	16.4 (3)	8.2 (8)	11.2 (2)	11.1 (3)	1.8 (1)	0.61 (7)	0.4 (1)	94.3 (7)	22 (2)	22 (1)	20 (1)	208 (3)
MLV12	28	52.5 (4)	1.0 (1)	18.3 (2)	4.3 (4)	8.9 (1)	12.2 (1)	1.93 (7)	0.70 (5)	0.08 (2)	96.0 (5)	8 (1)	13 (2)	12 (1)	224 (3)
MLV15	23	50.6 (4)	1.0 (1)	17.4 (2)	6.8 (6)	9.6 (2)	11.7 (2)	1.84 (7)	0.63 (5)	0.5 (1)	95.0 (5)	12 (2)	10 (2)	16 (3)	215 (1)
MLV16	22	50.2 (4)	0.9 (1)	17.1 (2)	7.5 (7)	9.7 (2)	11.6 (2)	1.78 (8)	0.61 (5)	0.54 (7)	94.4 (5)	13 (4)	12 (4)	18 (4)	212 (3)
MLV17	23	49.7 (4)	0.93 (8)	16.8 (2)	8.5 (5)	9.7 (2)	11.4 (2)	1.73 (7)	0.60 (5)	0.6 (1)	94.4 (4)	21 (8)	19 (8)	26 (5)	210 (2)
With decompression															
MLV03	76	49.9 (4)	0.9 (1)	17.2 (3)	7.8 (3)	9.9 (2)	11.4 (1)	1.81 (7)	0.63 (6)	0.18 (5)	96.7 (7)	30 (29)	30 (17)	22 (1)	213 (2)
MLV09	24	50.8 (4)	1.0 (1)	17.7 (3)	6.6 (3)	9.2 (4)	12.0 (2)	1.86 (5)	0.67 (6)	0.03 (1)	96.7 (7)	22 (6)	24 (8)	26 (2)	223 (2)
MLV14	24	49 (1)	0.9 (1)	17.2 (5)	9.8 (9)	9 (2)	11.4 (5)	1.7 (4)	0.63 (7)	0.18 (4)	96 (3)	54 (10)	64 (28)	49 (8)	213 (5)
MLV18	19	50.6 (3)	0.97 (8)	17.7 (2)	7.7 (5)	8.5 (2)	11.8 (2)	1.87 (7)	0.66 (5)	0.23 (7)	96.2 (6)	17 (3)	16 (2)	21 (3)	218 (2)
MLV19	23	49.9 (4)	0.9 (1)	17.3 (3)	8.9 (6)	8.9 (3)	11.5 (2)	1.80 (8)	0.65 (7)	0.21 (5)	96.2 (6)	35 (18)	36 (15)	31 (8)	212 (2)

*From Iacono-Marziano et al., 2017. n= number of analyses. [a] Anhydrous glass compositions by EMPA, normalised to 100. [b] Original total of the EMPA.

[c] Ni, Cu, Co and V contents analysed by LA-ICP-MS. Numbers in brackets are the standard deviation on the last decimal unit. bdl: below detection limit.

Supplementary Table 2. Sulphide droplet compositions

Sample	n	S [a] (wt%)	Fe [a] (wt%)	Ni [a] (wt%)	Cu [a] (wt%)	Co [a] (wt%)	O [a] (wt%)	Total [a] (wt%)
Without decompression								
MLV01	71	37.2 (6)	56.0 (7)	1.3 (2)	1.8 (2)	0.14 (1)	2.3 (2)	98.7 (8)
MLV02	19	36.0 (7)	55 (1)	1.8 (3)	3.0 (8)	0.15 (2)	2.2 (2)	98 (1)
MLV12	17	38.3 (6)	56 (1)	1.7 (4)	1.6 (9)	0.17 (1)	0.6 (4)	98.4 (4)
MLV15	23	37.4 (6)	56.3 (3)	1.4 (2)	1.8 (2)	0.13 (1)	1.8 (3)	98.8 (3)
MLV16	15	37.2 (8)	55.8 (6)	1.4 (3)	2.1 (7)	0.14 (2)	2.0 (3)	98.6 (3)
MLV17	18	36.7 (6)	55.4 (9)	1.6 (4)	2.5 (7)	0.15 (1)	2.3 (2)	98.7 (3)
With decompression								
MLV03	92	36 (1)	55 (1)	1.5 (3)	2.5 (5)	0.16 (1)	2.4 (7)	98 (1)
MLV09*	10	32 (1)	34 (2)	8 (2)	9 (2)	nd	nd	94 (1)
MLV14	17	35 (1)	51 (2)	3.0 (5)	6 (2)	0.21 (3)	2.3 (7)	98 (2)
MLV18	13	37 (1)	55.1 (8)	1.4 (1)	2.0 (3)	0.13 (1)	1.6 (6)	98 (1)
MLV19	31	37 (1)	52 (3)	2.5 (7)	4 (1)	0.17 (2)	1.9 (7)	98 (1)

n= number of analyses.

[a] Average composition of the sulfide melt by EMPA.

* Also contains 9 ± 2.5 wt% Pt. The Pt content of the other samples is < 1 wt%.

Numbers in brackets are the standard deviation on the last decimal unit.

Supplementary Table 3. Olivine composition

Sample	n	SiO_2 [a] (wt%)	FeO^{tot} [a] (wt%)	MgO [a] (wt%)	CaO [a] (wt%)	MnO [a] (wt%)	Total [a] (wt%)	Fo[b]	V[c] (ppm)
Without decompression									
MLV01	5	40.5 (6)	10.5 (8)	49.2 (6)	0.24 (4)	0.24 (6)	100.7 (8)	89.3 (8)	7 (2)
MLV02	7	41.8 (3)	9.8 (9)	48.2 (7)	0.25 (2)	0.22 (3)	100.3 (1)	89.8 (9)	5 (2)
MLV12	6	41.4 (2)	8.1 (6)	50.5 (5)	0.32 (2)	0.30 (4)	100.6 (5)	91.7 (6)	14 (1)
MLV15	5	40.9 (3)	10 (1)	49.0 (7)	0.24 (4)	0.35 (3)	100.2 (4)	90 (1)	7 (3)
MLV16	5	41.1 (4)	10.0 (6)	49.1 (6)	0.26 (7)	0.30 (4)	100.8 (6)	89.7 (6)	4 (1)
MLV17	5	40.6 (4)	11 (1)	48.2 (8)	0.22 (5)	0.28 (2)	100.4 (6)	89 (1)	3 (1)
With decompression									
MLV03	9	40.6 (4)	9.7 (5)	50.1 (4)	0.25 (3)	0.21 (7)	100.9 (8)	90.2 (4)	5.5 (6)
MLV09	7	41.4 (1)	9.3 (4)	49.7 (3)	0.21 (3)	0.26 (7)	100.9 (3)	90.5 (4)	5 (2)
MLV14	5	40.8 (4)	11.1 (4)	48.4 (4)	0.23 (3)	0.23 (9)	100.9 (5)	88.6 (3)	3 (1)
MLV18	5	41.0 (1)	10 (1)	48.9 (7)	0.25 (4)	0.27 (8)	100.6 (5)	90 (1)	3.3 (6)
MLV19	5	41.0 (6)	10.1 (4)	48.7 (2)	0.23 (2)	0.20 (9)	100 (1)	89.6 (4)	3.7 (7)

n= number of analyses. [a] Average composition of the olivine crystals by EMPA**.** [b] Forsterite content. [c] V content analysed by LA-ICP-MS.

Numbers in brackets are the standard deviation on the last decimal unit.

Supplementary Table 4. HRXCT statistics

Sulphide equivalent sphere diameter (μm)	Volume proportions (%)				Number of sulphide droplets per volume unit			
	MLV16	MLV17	MLV14	MLV19	MLV16	MLV17	MLV14	MLV19
0-20	0.82	1.07	0.74	0.79	1672.6	1680.2	281.0	406.1
21-40	6.81	6.96	3.82	3.53	1942.9	1625.1	259.9	134.4
41-60	10.34	8.59	5.23	3.53	604.0	400.0	71.4	20.7
61-80	14.84	11.46	6.62	4.86	317.7	193.2	35.3	5.8
81-100	16.88	11.24	6.65	4.04	172.6	87.9	15.9	2.2
101-120	13.56	13.35	9.18	3.30	76.6	57.0	11.5	3.0
121-140	11.51	13.25	8.45	1.85	37.7	34.3	6.3	3.3
141-160	6.87	8.13	7.00	3.31	15.4	14.0	3.6	3.7
161-180	4.39	4.89	7.98	8.82	6.9	5.8	2.8	6.6
181-200	6.56	1.77	2.83	2.79	6.9	1.4	0.8	1.5
201-220	2.18	2.28	3.75	1.68	1.7	1.4	0.8	0.7
221-240	1.87	1.92	6.23	4.78	1.1	1.0	0.8	1.5
241-260	0	1.47	10.21	3.20	0	0.5	1.2	0.7
261-280	1.42	0	0	2.14	0.6	0	0	0.4
281-300	1.96	0	11.69	2.23	0.6	0	0.8	0.4
301-320	0	2.58	0	0	0	0.5	0	0
321-340	0	2.97	0	3.58	0	0.5	0	0.4
341-360	0	3.80	9.63	0	0	0.5	0.4	0
361-380	0	4.25	0	4.93	0	0.5	0	0.4
381-400	0	0	0	0	0	0	0	0
401-420	0	0	0	13.19	0	0	0	0.7
421-440	0	0	0	0	0	0	0	0
441-460	0	0	0	9.16	0	0	0	0.4
461-480	0	0	0	0	0	0	0	0
481-500	0	0	0	0	0	0	0	0
501-520	0	0	0	0	0	0	0	0
521-540	0	0	0	0	0	0	0	0
541-560	0	0	0	0	0	0	0	0
561-580	0	0	0	18.28	0	0	0	0.4

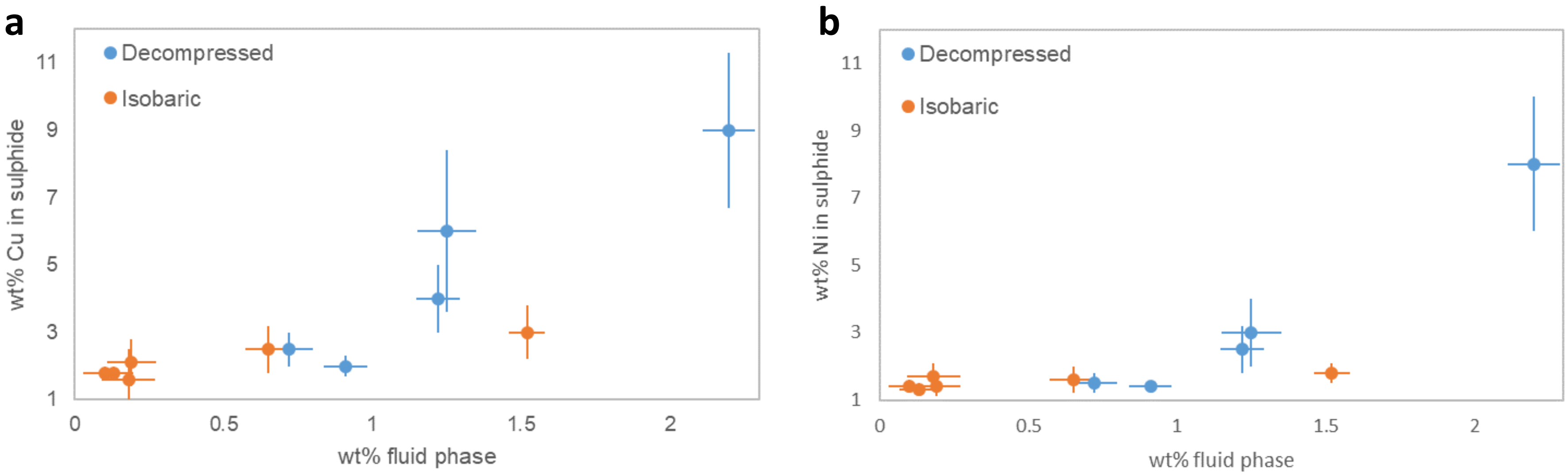

Supplementary Fig.1: Cu and Ni contents of the sulphide melt in experimental samples.

a Average Cu content of the sulphide melt versus the amount of fluid phase in each sample. **b** Average Ni content of the sulphide melt versus the amount of fluid phase in each sample. Error bars represent standard deviations around mean values.

Calculations of variations in Pt and Pd contents with silicate/sulphide mass ratio *R*

We used the equation of Campbell and Naldrett[10] to calculate how Pt and Pd contents of the sulphide melt vary when the amount of sulphide melt decreases (i.e., R-factor increases). We considered a parent magma with 10 ppb Pt and 10 ppb Pd, which are the average contents measured in the basaltic lavas of the Noril'sk region[2]. These contents are considered to represent Pt and Pd contents of the parental magma of ore-bearing intrusions, although higher contents have been analysed[2]. We used sulphide-silicate partition coefficients of either 10^4 or 10^5 both for Pt and Pd, in the range of those determined by ref. 30. Results are negligibly influenced by the choice of the partition coefficient (Supplementary Fig. 2), and are in a relatively good agreement with those obtained for Ni and Cu (Fig. 7): the average Pt contents of massive sulphides (64 analyses from

refs. 2 and 43) and those of disseminated sulphides (30 analyses recalculated to 100% sulphide, from refs. 2) are reproduced with an R-factor of 400 and 1300, respectively, whereas the average Pd contents are underestimated by a factor of less than 3 (Supplementary Fig. 2). The highest Pt and Pd contents observed in Noril'sk-Talnakh massive and globular ores are slightly underestimated by calculations with *R*=3000, whereas Ni and Cu contents are not (Fig.7). This suggest that PGM formation by sulphide melt consumption may contribute additional Pt and Pd to the amount supplied by the sulphide melt.

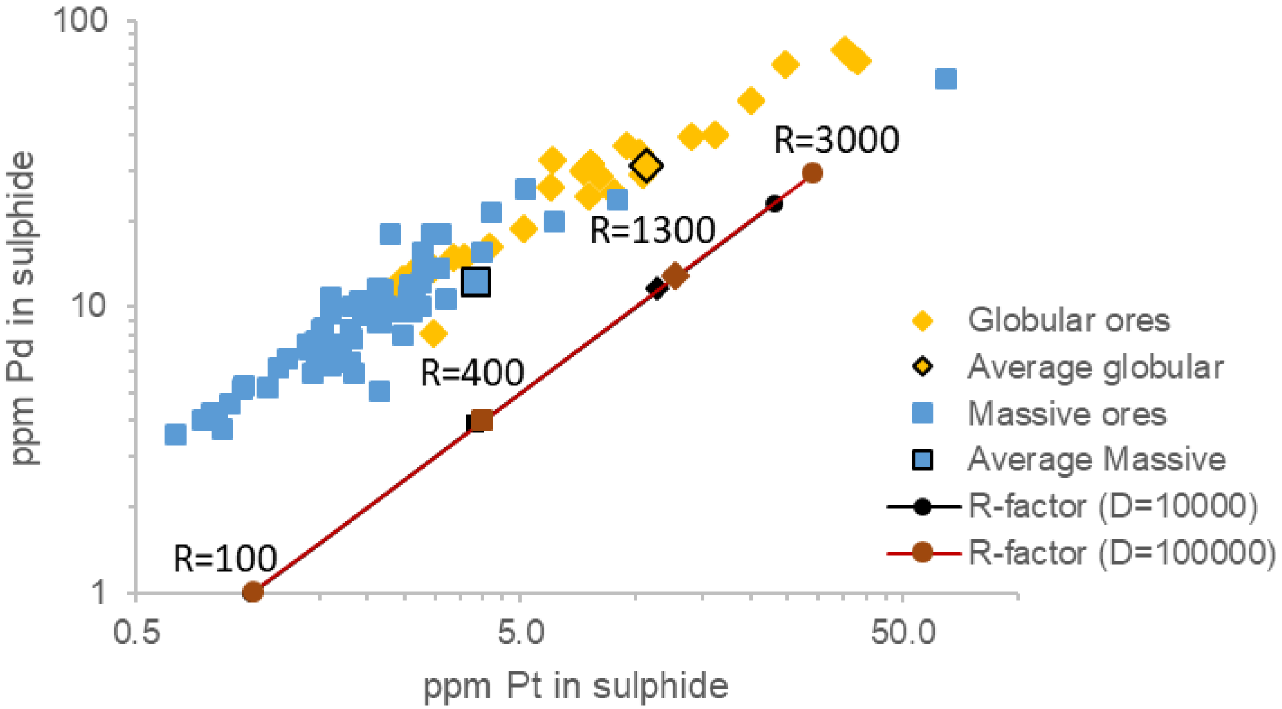

Supplementary Fig.2: Pt and Pd contents of globular and massive sulphide ores in Noril'sk-Talnakh intrusions. Data for globular sulphides are recalculated to 100% sulphide and are from ref. 2; the average of 30 analyses is shown. Data for massive sulphides are from refs. 2 and 43; the average of 64 analyses is shown. The solid line indicates equilibrium contents for variable silicate/sulphide mass ratios, i.e., *R* (indicated on the lines), calculated following Campbell and Naldrett's equation[10] using Dsulf/sil values of 10000 and 100000.
